# Supplementary figures and images for: Renal and major clinical outcomes and their determinants after nephrectomy in patients with pre-existing chronic kidney disease: A retrospective cohort study
Source: PLoS One. 2024 May 2;19(5):e0300367. doi: 10.1371/journal.pone.0300367 (PMC11065299; doi:10.1371/journal.pone.0300367)

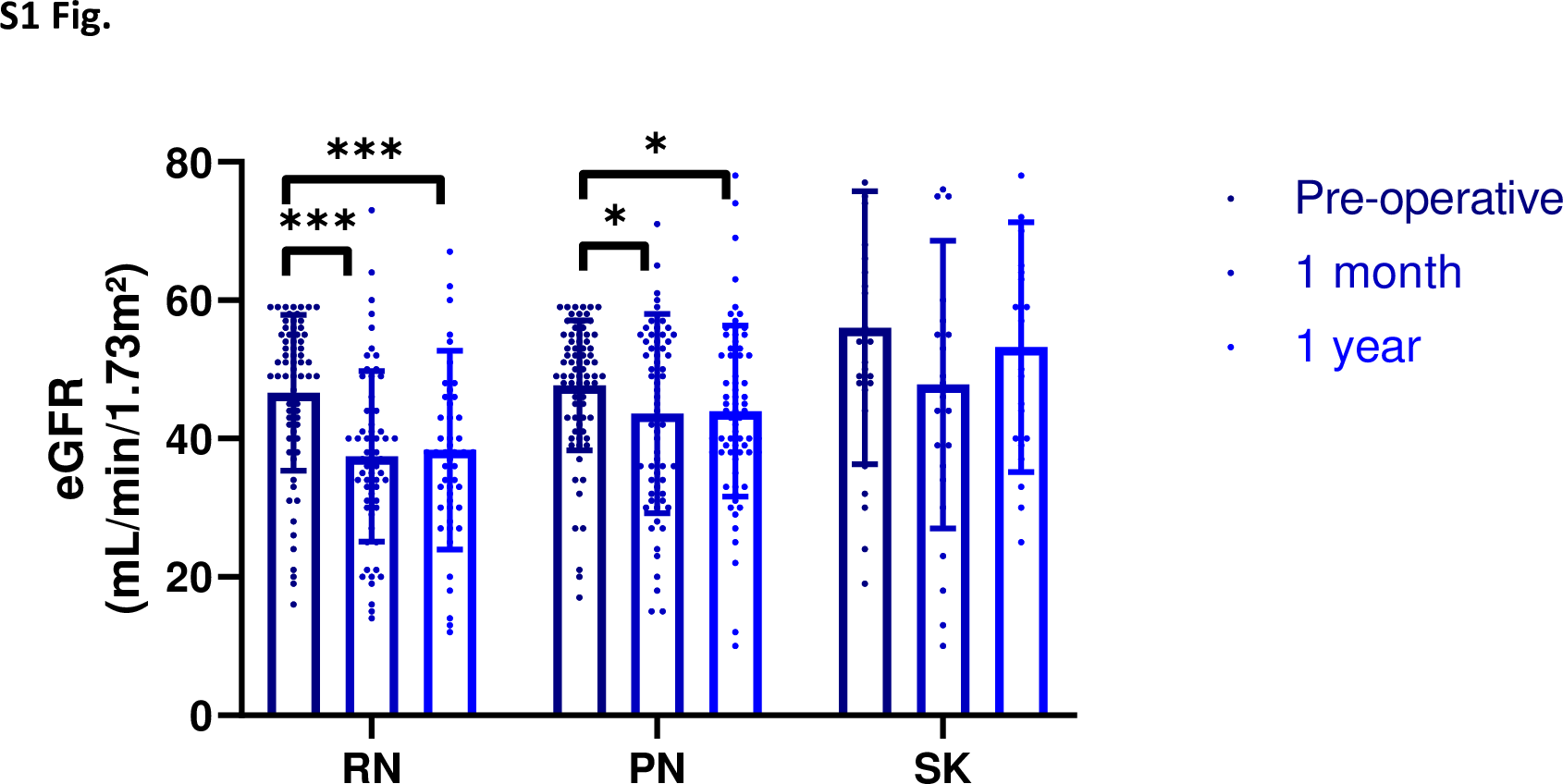

Supplement: S1 Fig — Data are presented as median with interquartiles. * p < 0.05; *** p < 0.001 (paired t-tests). eGFR estimated glomerular filtration rate, RN radical nephrectomy, PN partial nephrectomy, SK solitary kidney. (TIF) [file pone.0300367.s001.tif]

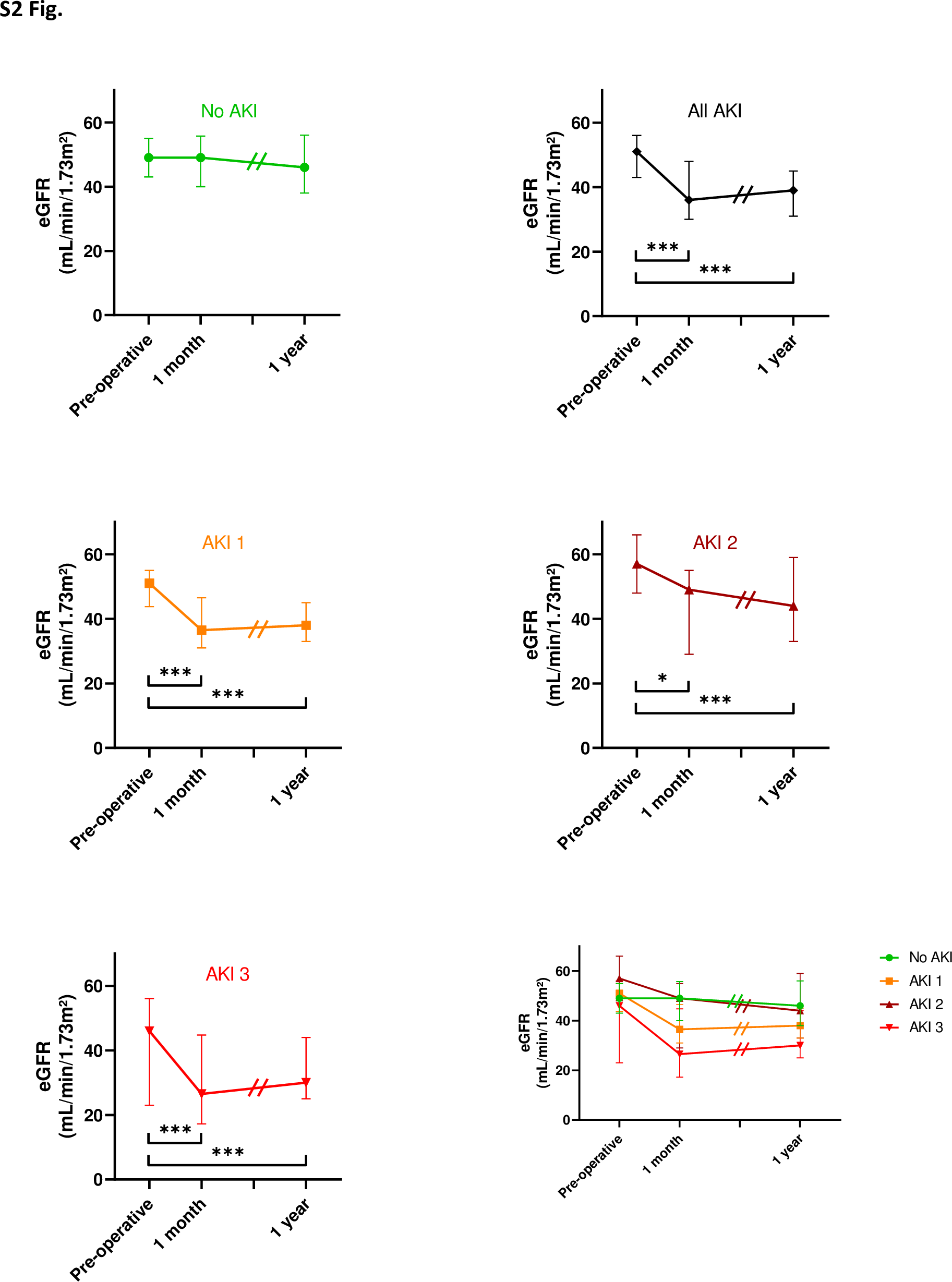

Supplement: S2 Fig — Data are presented as median with interquartiles. * p < 0.05; *** p < 0.001 (paired t-tests). eGFR estimated glomerular filtration rate, AKI acute kidney injury, KDIGO Kidney Disease: Improving Global Outcomes. (TIF) [file pone.0300367.s002.tif]
