# Supplementary material for: Renal and major clinical outcomes and their determinants after nephrectomy in patients with pre-existing chronic kidney disease: A retrospective cohort study
Source: PLoS One. 2024 May 2;19(5):e0300367. doi: 10.1371/journal.pone.0300367 (PMC11065299; doi:10.1371/journal.pone.0300367)
Supplement: S1 Table — Multivariable logistic regression and linear regression analysis investigating predictors of respectively CKD progression or of absolute the eGFR loss 1 year after surgery. AKI staging was defined according to KDIGO 2012 guidelines. CKD chronic kidney disease, AKI acute kidney injury, KDIGO Kidney Disease: Improving Global Outcomes, eGFR estimated glomerular filtration rate. (PDF) [file pone.0300367.s003.pdf]

S1 Table. Linear and logistic regression analysis for secondary outcomes

Model for CKD progression	Odds Ratio	95% CI	*p*-value
Age, for each additional year	1.021	0.971 – 1.074	0.414
Sex, female (ref) vs male	0.948	0.369 – 2.437	0.912
Diabetes mellitus, no (ref) vs yes	1.427	0.558 – 3.647	0.457
Hypertension, no (ref) vs yes	1.661	0.613 – 4.505	0.319
AKI, no (ref)			< 0.001
- AKI stage KDIGO 1	6.514	2.443 – 17.368	<0.001
- AKI stage KDIGO 2	11.112	1.764 – 70.012	0.01
- AKI stage KDIGO 3	31.502	5.632 – 176.209	<0.001
Tumor diameter, for each additional cm	1.189	1.002 – 1.411	0.047
Preoperative eGFR, for each additional mL/min			NS
Model for mean eGFR loss	Regression coefficient β		*p*-value
Age, for each additional year	- 0.104		0.224
Sex, female (ref) vs male	0.114		0.171
Diabetes mellitus, no (ref) vs yes	- 0.104		0.226
Hypertension, ne (ref) vs yes	0.061		0.478
AKI, no (ref) vs yes	- 0.359		< 0.001
Tumor diameter, for each additional cm	- 0.215		0.013
Preoperative eGFR, for each additional mL/min			NS

Multivariable logistic regression and linear regression analysis investigating predictors of respectively CKD progression or of absolute the eGFR loss 1 year after surgery.

AKI staging was defined according to KDIGO 2012 guidelines.

CKD chronic kidney disease, *AKI* acute kidney injury, *KDIGO* Kidney Disease: Improving Global Outcomes, *eGFR* estimated glomerular filtration rate.
