# Supplementary material for: Renal and major clinical outcomes and their determinants after nephrectomy in patients with pre-existing chronic kidney disease: A retrospective cohort study
Source: PLoS One. 2024 May 2;19(5):e0300367. doi: 10.1371/journal.pone.0300367 (PMC11065299; doi:10.1371/journal.pone.0300367)
Supplement: S2 Table — Data are presented as median with interquartile [IQR] or number and frequencies (%). CKD chronic kidney disease, SK solitary kidney, RN radical nephrectomy, PN partial nephrectomy, MACE major adverse cardio-vascular outcome, eGFR estimated glomerular filtration rate. (PDF) [file pone.0300367.s004.pdf]

**S2 Table. One-month postoperative renal outcomes**

| | CKD patients | | | *p*-value | SK patients | *p*-value | |
|---|---|---|---|---|---|---|---|
| | All-CKD (*n* = 146) | RN-CKD (*n* = 67) | PN-CKD (*n* = 79) | | PN-SK (*n* = 27) | vs CKD-RN | vs CKD-PN |
| 1-month primary outcome (%) | 61/129 (47) | 36/63 (57) | 25/66 (38) | 0.03 | 18/27 (67) | NS | 0.01 |
| 1-month CKD progression (%) | 59/129 (46) | 36/62 (58) | 23/67 (34) | 0.007 | 17/26 (65) | NS | 0.007 |
| 1-month MACE (%) | 5/137 (4) | 2/66 (3) | 3/71 (4) | NS | 1/27 (4) | NS | NS |
| 1-month mortality (%) | 1/137 (1) | 1/66 (2) | 0/71 | NS | 1/27 (4) | NS | NS |
| | | | | | | | |
| 1-month eGFR mL/min/1.73m² | 40 [32-51] | 37 [30-44] | 43 [33-54] | 0.01 | 47 [35-59] | 0.01 | NS |
| 1-month eGFR loss mL/min/1.73m² | 6 [0-16] | 12 [3-17] | 3 [0-11] | <0.001 | 6 [0-18] | NS | NS |
| 1-month eGFR loss % of baseline | 15 [0-31] | 25 [6-35] | 6 [0-25] | <0.001 | 13 [1-35] | NS | NS |
| 1-month eGFR <90% of baseline | 76/129 (59) | 46/62 (74) | 30/67 (45) | <0.001 | 16/26 (62) | NS | NS |
| 1-month on hemodialysis (%) | 2/137 (1) | 2/66 (3) | 0/71 | NS | 1/26 (4) | NS | NS |
| 1-month known nephrology referral (%) | 41/128 (32) | 17/61 (28) | 24/67 (36) | NS | 19/26 (73) | <0.001 | 0.001 |

Data are presented as median with interquartile [IQR] or number and frequencies (%).

*CKD* chronic kidney disease, *SK* solitary kidney, *RN* radical nephrectomy, *PN* partial nephrectomy, *MACE* major adverse cardio-vascular outcome, *eGFR* estimated glomerular filtration rate.
