# Supplementary material for: Renal and major clinical outcomes and their determinants after nephrectomy in patients with pre-existing chronic kidney disease: A retrospective cohort study
Source: PLoS One. 2024 May 2;19(5):e0300367. doi: 10.1371/journal.pone.0300367 (PMC11065299; doi:10.1371/journal.pone.0300367)
Supplement: Attachment — Submitted filename: Response to Reviewers.docx [file pone.0300367.s006.docx]

Doctor Yudai Ishiyama,

Academic Editor,

PLOS ONE

November 26^th^, 2023

Dear Dr. Ishiyama,

We thank the reviewers and academic editor for their valuable comments.

Please find appended the revised version of the manuscript entitled “Renal and major clinical outcomes and their determinants after nephrectomy in patients with pre-existing chronic kidney disease: a retrospective cohort study” (one version with modifications made apparent, and a second clean version), as well as a point-by-point reply to comments made by the academic editor and reviewers.

We hope that this version is suitable for publication, but remain at your disposal if any further modifications are required.

Thank you for your consideration,

Yours sincerely,

Dr Maxime Schleef, on behalf of all authors

**Journal requirements:**

1. We ensured carefully that our manuscript meets PLOS ONE’s style requirements, and we hope this version is acceptable for this regard.

2. We uploaded an anonymized data set as a supporting information file as requested, and we appreciate your proposition of updating the data availability statement on our behalf.

**Additional Editor Comments, Comment 1:** First, the authors’ decision to include single-kidney patients regardless of their CKD status seems illogical and makes their message vague and unsolid. They should either exclude this population or provide a clearer reason for its inclusion.

**Authors’ reply:** Our decision to include solitary kidney (SK) patients regardless of their chronic kidney disease (CKD) status may seem counter-intuitive at first, but we believe it can provide valuable information and that this population should not be excluded. SK patients were often excluded in previous studies focusing on outcomes after partial nephrectomy, and therefore analyzed in their own separate studies, and data about these patients are scarce. In the present study, we wanted to focus specifically on CKD patients to test it their outcomes after nephrectomy were consistent with what has been reported in general population, with patients mostly with a normal renal function, and usually with the exclusion of severe CKD.

Foremost, SK condition in itself is, and should be, considered a CKD, and recognized as such even when impairment of glomerular filtration rate (GFR) has not occurred yet. In Kidney Disease: Improving Global Outcomes (KDIGO) guidelines (Kidney International Supplements (2013) 3, 5–14), CKD is defined as chronic abnormalities of kidney function (GFR < 60 mL/min/1.73m²) OR kidney structure as well, including structural abnormalities detected by imaging such as anatomical or functional solitary kidney. Even when GFR remains normal, SK should therefore be classified as CKD stage 1-2, reflecting the increased susceptibility of renal complications such as acute kidney injury, GFR deterioration, and CKD progression, and in order to emphasize the importance of nephroprotection. Having a SK, even with normal renal function, has indeed been proved as an independent risk factor of renal function deterioration leading to progression of CKD (Kim et al., 2019, Eur J Epidemiol, doi: 10.1007/s10654-019-00520-7).

Likewise, American Urological Association (AUA) and European Association of Urology (EAU) guidelines (respectively, doi:10.1016/j.juro.2017.04.100 and doi:10.1016/j.eururo.2019.02.011) also associate SK patients regardless of renal function along with patients with renal dysfunction (moderate to severe CKD), as they classify them similarly as having an “imperative” indication of nephron sparing surgery (as opposed to an “elective” indication) to preserve their renal parenchyma and GFR. For example, in the RECORd1 cohort, patients presenting with an imperative indication of PN, including moderate to severe CKD and SK regardless of renal function, were analyzed together in the same subgroup (Minervini et al. 2019, Minerva Urologica E Nefrologica, doi:10.23736/S0393-2249.18.03202-2). In this cohort, imperative surgery (that is patients with CKD and SK regardless of renal function) was a significant predictive risk factor of intra-operative complications, followed by higher risk of overall postoperative complications.

Furthermore, among the 11 SK patients with CKD stage 1-2 in our cohort, it is noteworthy that 8 (72%) of them experienced post-operative AKI. Consequently, 7 (63%) of them also presented the primary outcome at 1 year, with a progression of CKD, which is a serious and concerning event in this population. This emphasizes their great susceptibility to renal lesions during and after partial nephrectomy, in the short and long term, precipitating them from CKD stages with normal to impaired functions, and ultimately exposing them to potential complications due to CKD.

For all these reasons, we are convinced that all SK patients, even with CKD stage 1-2, are a valuable addition to this paper and its conclusion, and their inclusion provide useful data for practitioners managing renal tumor. The importance of postoperative acute kidney injury as a predictive risk factor of poor renal outcomes should be particularly concerning in daily practice, especially with SK patients as showed by its high incidence in our cohort, and a normal renal function in SK patients should not be falsely reassuring in their preoperative outcome. We believe that the exclusion of SK patients with CKD stage 1-2 would convey a wrong message.

As requested, we detailed more these reasons in the discussion section of the manuscript as follow: “Furthermore, the cohort herein is one of the largest comparing PN and RN specifically in patients with moderate-to-severe pre-existing CKD, alongside patients with SK. It is noteworthy that we included SK patients regardless of their CKD staging, including a few patients with stage 1 and 2, i.e. even with eGFR > 60 mL/min/1.73m². This was motivated by the fact that SK condition in itself is considered a CKD, even when impairment of eGFR has not occurred yet (30). Having a SK, even with normal renal function, has indeed been proved as an independent risk factor of renal function deterioration leading to progression of CKD (31). Likewise, American Urological Association (AUA) and European Association of Urology (EAU) guidelines also associate SK patients regardless of renal function along with patients with renal dysfunction (moderate to severe CKD), as they classify them similarly as having an “imperative” indication of nephron sparing surgery (2,3). In the present study, the high incidence of AKI and of the primary outcome in SK patients emphasizes their great susceptibility to renal lesions during and after partial nephrectomy, in the short and long term. Precipitating SK patients from CKD stages with normal to impaired functions is a particularly concerning event, as this will ultimately exposes them to potential complications due to CKD.”.

**Additional Editor Comments, Comment 2:** Second, if surgical method (radical vs partial) could not be included in the multivariable model due to a strong correlation with tumor size, the statement ‘PN was associated with lower risk of CKD progression, MACE, or death, one year after surgery, compared to RN’ is an overstatement, especially in the abstract.

**Authors’ reply:** Surgical technique could indeed not be included in the multivariate model due to collinearity with tumor size, as it would have been statistically inappropriate. We understand the editor’s concern about this statement, so we modified the abstract and deleted the following statement “~~In moderate-to-severe CKD patients, PN was associated with lower risk of~~ ~~CKD progression, MACE, or death, one year after surgery, compared to RN.~~ Postoperative AKI after PN or RN was the major independent determinant of worse outcomes (CKD progression, MACE, or death) one year after surgery.” and the conclusion of the manuscript was modified as follow: “In conclusion, we report that in patients with moderate to severe CKD, postoperative AKI was a major independent predictor of the composite outcome including CKD progression, MACE, or death, one year after surgery. Further studies are needed to identify early determinants of AKI in this population. PN led to better outcomes compared to RN, but these results need to be confirmed as the type of surgery could not be integrated in multivariate analysis.”.

**Reviewer 1, Comment 1:** First, please show us the criteria for choosing PN or RN in author's institution. I want to know author's entire clinical records of preoperative clinical T stage, median tumor size for calculating using CT scan.

**Authors’ reply:**

The criteria for choosing PN or RN was based on french guidelines at the time (doi: 10.1016/S1166-7087(13)70055-1 and doi: 10.1016/S1166-7087(16)30702-3), in which PN was the first option whenever feasible, similar to and still in accordance with current french, european and american guidelines (respectively, doi: 10.1016/S1166-7087(20)30749-1, doi: 10.1016/j.eururo.2019.02.011, and doi: 10.1016/j.juro.2017.04.100). We therefore added in the methods section of the manuscript the following statement: “Inclusion criteria were any surgical procedure (RN or PN) for a renal tumor from January 2013 to December 2018, with a baseline estimated glomerular filtration rate (eGFR) < 60 mL/min/1.73m² prior to surgery (RN-CKD and PN-CKD groups), or a PN for a renal tumor on a SK regardless of eGFR (PN-SK group). The criteria for choosing PN or RN were based on french guidelines at the time, in which PN was the first option whenever feasible (26).”.

Tumor size showed in Table 2 in the manuscript was measured on pathological exam report after the tumor was removed. Tumor size according to CT scan evaluation was also available, whenever it was reported in surgeons’ preoperative consultations (however, we did not have direct acces to CT scan images, CT scan reports results), so we evaluated pre operative clinical T stage based on these informations with the following results:

|  | CKD patients (*n* = 146) | | *p*-value | SK patients | *p*-value | | |
| --- | --- | --- | --- | --- | --- | --- | --- |
|  | RN-CKD (*n* = 67) | PN-CKD (*n* = 79) |  | PN-SK (*n* = 27) | vs RN-CKD | vs PN-CKD | |
| Tumor length on preoperative imaging, mm | 70 (44-99) | 36 (26-52) | <0.001 | 35 (22-50) | <0.001 | NS | |
| cTNM staging (%): |  |  |  |  |  |  |  |
| - T1 | 18 (27) | 68 (86) | <0.001 | 23 (85) | <0.001 | NS |  |
| - T2 | 13 (19) | 7 (9) | NS | 1 (4) | NS | NS |  |
| - T3 | 22 (33) | 0 | <0.001 | 1 (4) | 0.003 | NS |  |
| - T4 | 0 | 0 | NA | 0 | NA | NA |  |
| - Tx | 14 (21) | 4 (5) | 0.004 | 2 (7) | NS | NS |  |

These results are very similar to those we presented in Table 2. We believe that Table 2 already contains a lot of informations, but if the Editor would like us to do so, we suggest to add it to Table 2.

**Reviewer 1, Comment 2:** Second, I do not understand for including SK patients with CKD1-2 stage. If authors analyze the comparison of renal outcomes between PN and RN in CKD≥3 patients, you do not have to compare for three groups.

**Authors’ reply:** Our decision to include solitary kidney (SK) patients regardless of their chronic kidney disease (CKD) status may seem counter-intuitive at first, but we believe it can provide valuable information and that this population should not be excluded. SK patients were often excluded in previous studies focusing on outcomes after partial nephrectomy, and therefore analyzed in their own separate studies, and data about these patients are scarce. In the present study, we wanted to focus specifically on CKD patients to test it their outcomes after nephrectomy were consistent with what has been reported in general population, with patients mostly with a normal renal function, and usually with the exclusion of severe CKD.

Foremost, SK condition in itself is, and should be, considered a CKD, and recognized as such even when impairment of glomerular filtration rate (GFR) has not occurred yet. In Kidney Disease: Improving Global Outcomes (KDIGO) guidelines (Kidney International Supplements (2013) 3, 5–14), CKD is defined as chronic abnormalities of kidney function (GFR < 60 mL/min/1.73m²) OR kidney structure as well, including structural abnormalities detected by imaging such as anatomical or functional solitary kidney. Even when GFR remains normal, SK should therefore be classified as CKD stage 1-2, reflecting the increased susceptibility of renal complications such as acute kidney injury, GFR deterioration, and CKD progression, and in order to emphasize the importance of nephroprotection. Having a SK, even with normal renal function, has indeed been proved as an independent risk factor of renal function deterioration leading to progression of CKD (Kim et al., 2019, Eur J Epidemiol, doi: 10.1007/s10654-019-00520-7).

Likewise, American Urological Association (AUA) and European Association of Urology (EAU) guidelines (respectively, doi:10.1016/j.juro.2017.04.100 and doi:10.1016/j.eururo.2019.02.011) also associate SK patients regardless of renal function along with patients with renal dysfunction (moderate to severe CKD), as they classify them similarly as having an “imperative” indication of nephron sparing surgery (as opposed to an “elective” indication) to preserve their renal parenchyma and GFR. For example, in the RECORd1 cohort, patients presenting with an imperative indication of PN, including moderate to severe CKD and SK regardless of renal function, were analyzed together in the same subgroup (Minervini et al. 2019, Minerva Urologica E Nefrologica, doi:10.23736/S0393-2249.18.03202-2). In this cohort, imperative surgery (that is patients with CKD and SK regardless of renal function) was a significant predictive risk factor of intra-operative complications, followed by higher risk of overall postoperative complications.

Furthermore, among the 11 SK patients with CKD stage 1-2 in our cohort, it is noteworthy that 8 (72%) of them experienced post-operative AKI. Consequently, 7 (63%) of them also presented the primary outcome at 1 year, with a progression of CKD, which is a serious and concerning event in this population. This emphasizes their great susceptibility to renal lesions during and after partial nephrectomy, in the short and long term, precipitating them from CKD stages with normal to impaired functions, and ultimately exposing them to potential complications due to CKD.

For all these reasons, we are convinced that all SK patients, even with CKD stage 1-2, are a valuable addition to this paper and its conclusion, and their inclusion provide useful data for practitioners managing renal tumor. The importance of postoperative acute kidney injury as a predictive risk factor of poor renal outcomes should be particularly concerning in daily practice, especially with SK patients as showed by its high incidence in our cohort, and a normal renal function in SK patients should not be falsely reassuring in their preoperative outcome. We believe that the exclusion of SK patients with CKD stage 1-2 would convey a wrong message.

As requested, we detailed more these reasons in the discussion section of the manuscript as follow: “Furthermore, the cohort herein is one of the largest comparing PN and RN specifically in patients with moderate-to-severe pre-existing CKD, alongside patients with SK. It is noteworthy that we included SK patients regardless of their CKD staging, including a few patients with stage 1 and 2, i.e. even with eGFR > 60 mL/min/1.73m². This was motivated by the fact that SK condition in itself is considered a CKD, even when impairment of eGFR has not occurred yet (30). Having a SK, even with normal renal function, has indeed been proved as an independent risk factor of renal function deterioration leading to progression of CKD (31). Likewise, American Urological Association (AUA) and European Association of Urology (EAU) guidelines also associate SK patients regardless of renal function along with patients with renal dysfunction (moderate to severe CKD), as they classify them similarly as having an “imperative” indication of nephron sparing surgery (2,3). In the present study, the high incidence of AKI and of the primary outcome in SK patients emphasizes their great susceptibility to renal lesions during and after partial nephrectomy, in the short and long term. Precipitating SK patients from CKD stages with normal to impaired functions is a particularly concerning event, as this will ultimately exposes them to potential complications due to CKD.”.

**Reviewer 1, Comment 3:** Finally, it would be better to include postoperative taking ACEi/ARBs or nephtotoxic agents as a factor in the multivariable analysis.

**Authors’ reply:** We were indeed limited by the number of variables that could reasonably be included in the multivariate analysis, given the limitied size of our cohort (still one of the main contemporary cohort on this specific setting of CKD) and to stay statistically sound. We tried to integrate in priority variables that had previously been described as independent risk factors of poor outcomes. Nephroprotective medications such as ACEi and ARBs, and the control of proteinuria, are also known cornerstones of long term renal function preservation in CKD, among others (nephrotoxic agents eviction, cardiovascular risk factors prevention…) and could have been valuable covariates as reviewer 1 rightfully proposed it. Unfortunately, these data could not be obtained in our retrospective cohort. Only anesthesiologic, surgical and hospitalization records (demographic, past medical history, tumor and surgery characteristics, complications during hospitalization…) were accessible, as well as data on lab tests of renal function (serum creatinin and estimated glomerular filtration) prior to and after surgery, however details on nephrology follow-up (including nephroprotective medications and interventions, urinalysis…) were not available on the electronic medical record held by anesthesiologists and surgeons. We acknowledged these limitations in the discussion section as follow: “we could not take into account other unknown confounders, or known but unavailable, such as the presence of proteinuria on the risk of CKD progression (39) and use of ACEi/ARBs, or post-operative use of contrast or other nephrotoxic agents”.

**Rewiever 2, Comment:** The study appears to be well-conducted with a clear methodology and relevant outcomes. The results provide valuable insights into the effects of renal surgeries on CKD patients. However, as with all retrospective studies, there's potential for selection bias and reliance on the accuracy of previously recorded data. It would be beneficial to see a prospective study or randomized controlled trial in the future to validate these findings further. Additionally, the generalizability of the results might be limited to the specific population and setting of the study. Overall, the study adds valuable knowledge to the field and has potential implications for clinical practice.

**Authors:** We thank reviewer 2 for his positive review on our work. We indeed acknowledge the main limits of our study being its retrospective design (as we did in the discussion section), with its inherent potential bias, but also its monocentric setting, as reviewer 2 rightfully evoked it, so we added it in the discussion. We nevertheless hope that the conclusions brought by this work will further raise awareness on the greater susceptibility of CKD and SK patients to acute kidney injury and CKD progression after nephrectomy, and the need for new supportive therapeutics, and will create dynamics to conduct well-designed prospective studies following this specific population of patients, hopefully eventually leading to interventional trials assessing strategies of nephroprotection in this setting. In any cases, we detailed the main limitations of our study more precisely in the discussion, as suggested: “The present study has limitations. Due to its retrospective nature, we cannot rule out selection bias, and bias in data collection. Its monocentric setting limits the generalizability of the results in other different population of CKD patients undergoing nephrectomy.”.
