# Supplementary material for: Renal and major clinical outcomes and their determinants after nephrectomy in patients with pre-existing chronic kidney disease: A retrospective cohort study
Source: PLoS One. 2024 May 2;19(5):e0300367. doi: 10.1371/journal.pone.0300367 (PMC11065299; doi:10.1371/journal.pone.0300367)
Supplement: Attachment — Submitted filename: Response to Reviewers.docx [file pone.0300367.s007.docx]

Doctor Yudai Ishiyama,

Academic Editor,

PLOS ONE

February 20^th^, 2024

Dear Dr. Ishiyama,

We thank the editor for the additional comments that help us improve the quality of our manuscript.

Please find appended the revised version of the manuscript entitled “Renal and major clinical outcomes and their determinants after nephrectomy in patients with pre-existing chronic kidney disease: a retrospective cohort study” (one version with modifications made apparent, and a second clean version), as well as a point-by-point reply to the comments.

We hope that this version is suitable for publication, but then again we remain at your disposal if any further modifications are necessary.

Thank you for your time and consideration,

Yours sincerely,

Dr Maxime Schleef, on behalf of all authors

**Additional Editor Comments, Comment #1:** The initial review did not mention this, but it seems a significant portion of patients in the RN-CKD cohort had a final diagnosis of urothelial carcinoma. It's important to confirm whether the surgical procedure intended from start to finish for these patients was radical nephrectomy, not nephroureterectomy.

**Authors’ reply:** About ¼ of the patients in the RN-CKD group indeed had a final diagnosis of urothelial carcinoma on the pathological exam report. As we explained in the Methods section, we included surgical procedures intended for a renal tumor only (as reported by our surgeons according to the French Hospital Discharge Summaries Database), and of course we did not consider procedures planned for a urinary excretory system or urinary tract tumor. It is however plausible that in these few cases, the tumors were thought to be developing at the expense of the renal parenchyma, close to the pyelic cavities, and later found that those were rather urothelial tumors. The surgeries were still classified in the end “radical nephrectomies” by surgeons, eventually because it was the surgical procedure intended from start to finish.

**Additional Editor Comments, Comment #2:** It's also worth noting that no patients with urothelial carcinoma were included in the PN-CKD or SK groups. Among those who died of cancer within one year, how many had urothelial carcinoma pathology? If the majority of deceased patients had urothelial carcinoma rather than RCC, comparing these groups might seem inappropriate.

**Authors’ reply:** Of the 67 RN-CKD patients, 7 died within one year after surgery. Among them, 3 had a final diagnosis of urothelial carcinoma (about 17% of the 18 patients with urothelial carcinoma): 1 death due to a multi-metastatic evolution (noteworthy the patient had started hemodialysis in-between), 1 death due to a cerebral hemorrhage, 1 death at home due to an unknown cause (it is worth noting that in-between this patient had started hemodialysis, had presented an episode of pulmonary edema and an episode of unstable angina). The 4 other remaining patients had a final diagnosis of renal cell carcinoma (about 11% of the 38 patients with RCC in RN-CKD): 2 deaths due to pulmonary metastasis, 2 deaths due to pulmonary embolism (including one with a vena cava thrombosis).

In comparison, of the 27 PN-SK patients, one died due to aspiration pneumonia secondary to bowel obstruction, and of the 79 PN-CKD patients, one died due to a multi-metastatic evolution; both patients had a final diagnosis of renal cell carcinoma.

We understand the general concern about the comparability of the RN and PN groups, as we chose to include surgeries performed for any type of renal tumor, and not only renal cell carcinomas, and we highlighted it in the discussion section. We chose foremost to investigate the effect of these procedures, regardless of the underlying oncological disease, on renal function, notably through the nephronic reduction, which is minimized by PN although it leads to ischemia-reperfusion injury due to the necessary vascular clamping. As already mentioned in the first round of review, to conclude that PN is better than RN based only on our data could indeed be considered an overstatement. We would rather emphasize the fact that these types of surgeries have deleterious effects on renal function and outcomes, even more in this specific population of CKD patients, but still probably minimized by PN. Another strong message that we try to convey is that postoperative acute kidney injury is highly prevalent in this population and is a strong independent predictor of worse outcomes, and effort should be made to find mean to prevent it.

**Additional Editor Comments, Comment #3:** Additionally, how many patients met both criteria for:

A) “1-year cancer-related mortality” and “1-year CKD progression,” or

B) “1-year cancer-related mortality” and “1-year MACE”?

If the majority of patients who died of cancer within a year met either criterion A or B, the statistical impact of the current analysis would be substantially affirmed.

**Authors’ reply:** Of the entire cohort (173 patients), only 9 patients died, including 5 patients who died of cancer within one year. One had already met CKD progression criteria (and had actually started hemodialysis), none of them had already presented a MACE, with respect to the information available during the retrospective collection of the data.

As stated before, one patient died at home due to an unknown cause but had already started hemodialysis, had presented an episode of pulmonary edema and an episode of unstable angina. The other patients that died from a non-cancer related cause (or were not proven so), had not presented CKD progression or MACE. To note, pulmonary embolism was not defined as MACE in our study.

Eventually, the mortality event was uncommon (9/173 patients (5%)) in the study herein which is underpowered to identify causality or draw any strong conclusion from this event.

**Additional Editor Comments, Comment #4:** Table 2 indicates that a certain proportion of patients received adjuvant systemic therapies. Please provide specific details (regimens) of these therapies, as some drugs may have nephrotoxic effects.

**Authors’ reply:** 8 patients received adjuvant systemic therapies, described thereafter:

|  | CKD patients (*n* = 146) | | *p*-value | SK patients | *p*-value | |
| --- | --- | --- | --- | --- | --- | --- |
|  | RN-CKD (*n* = 67) | PN-CKD (*n* = 79) |  | PN-SK (*n* = 27) | vs RN-CKD | vs PN-CKD |
| … |  |  |  |  |  |  |
| Adjuvant chemotherapy (%) | 6 (9) | 1 (1) | NS | 1 (4) | NS | NS |
| *- Sunitinib* | *2 (3)* | *0* |  | *0* |  |  |
| *- Pazopanib* | *1 (1)* | *0* |  | *1 (4)* |  |  |
| *- Temsirolimus* | *1 (1)* | *0* |  | *0* |  |  |
| *- Gemcitabine* | *1 (1)* | *1 (1)* |  | *0* |  |  |
| *- Unknown* | *1 (1)* | *0* |  | *0* |  |  |

Most of these patients (5) received anti-angiogenic/anti-VEGF therapies, 1 received m-TOR inhibitor, and 2 received Gemcitabine. The exact treatment was unknown in one patient, that was referred to another oncological center closer to his home, and the only reports subsequently found specified that the patient was receiving adjuvant chemotherapy with no precision. All these treatments are known for a rather good tolerance profile on renal function in general, although it is true that they can induce nephrotoxicity in rare cases (thrombotic microangiopathy, hypertension and proteinuria have for example been described). Noteworthy, among these 8 patients, only 3 (38%) met the CKD progression criteria within one year (1 on Temsirolimus, 1 on Sunitinib and 1 on Pazopanib), as opposed to respectively 63%, 36% and 52% of CKD progression in RN-CKD, PN-CKD and PN-SK in general. We found it reasonable to assume that the influence of these adjuvant systemic therapies on renal function, and on the results and conclusion of our study was negligible. If the editor would like us to do so, we can add a phrase to discuss this limitation in the discussion section, such as: “A few patients received adjuvant systemic therapies, which may rise concern about their nephrotoxic effects. However, among them the incidence of CKD progression criteria within one year was not higher than in the rest of the patients, it is therefore reasonable to assume that the influence of these adjuvant systemic therapies on renal function, and on the results and conclusion of our study was negligible”, and/or we can add these details in a supplementary file.
